# Comment on: Gaps and barriers in the implementation and functioning of antimicrobial stewardship programmes: results from an educational and behavioural mixed methods needs assessment in France, the United States, Mexico and India

**DOI:** 10.1093/jacamr/dlad050

**Published:** 2023-04-28

**Authors:** Martha Carolina Valderrama-Rios, Laura Cristina Nocua-Báez, Carlos Arturo Álvarez-Moreno, Jorge Alberto Cortes

**Affiliations:** Department of Internal Medicine, School of Medicine, Universidad Nacional de Colombia, Oficina de Investigación Clínica, Calle 44 no. 59-75, Primer piso, Bogotá, Colombia; Department of Internal Medicine, School of Medicine, Universidad Nacional de Colombia, Oficina de Investigación Clínica, Calle 44 no. 59-75, Primer piso, Bogotá, Colombia; Infectious Diseases Service, Hospital Universitario Nacional, Bogotá, Colombia; Department of Internal Medicine, School of Medicine, Universidad Nacional de Colombia, Oficina de Investigación Clínica, Calle 44 no. 59-75, Primer piso, Bogotá, Colombia; Department of Internal Medicine, School of Medicine, Universidad Nacional de Colombia, Oficina de Investigación Clínica, Calle 44 no. 59-75, Primer piso, Bogotá, Colombia

We have read the recent article published in the *Journal* by P. Lazure *et al*. on the barriers in the implementation and functioning of antimicrobial stewardship programmes (AMS),^1^ and we would like to congratulate the authors for this interesting article and make some complementary contributions to this important topic from our experience in a university hospital in a Latin American country.

In the article, it was indicated that the lack of knowledge and skills pertaining to best practices in the diagnosis of infections and antibiotic prescription is one of the existing barriers to the suitable functioning of AMS, concluding on the need to work on the education of health professionals involved in antibiotic prescribing, especially in countries where AMS may not be fully implemented. And, although we agree, we believe it is important to keep in mind an additional aspect that is not mentioned in the study by Lazure *et al*. and that may play an individual-level significant role during antibiotic prescription in the clinical practice: It is the fear of patient deterioration or complications.

At the Hospital Universitario Nacional de Colombia, a referral centre of high complexity in Bogotá (capital city of 8 million inhabitants), as part of the continual training activities for health professionals within the framework of the AMS, we developed a virtual course and a mobile application with the main recommendations of diagnosis and antibiotic prescription of the most prevalent infections.^2–4^ However, we identified that some antibiotic prescriptions were not concordant with the guidelines, due to improper antibiotic, dose, interval or duration. During feedback sessions by the infectious diseases staff, a survey was conducted that asked about the reason for these prescriptions.

The survey was carried out during four consecutive months, from 1 June to 30 September 2022, and feedback was provided to healthcare professionals who prescribed an antibiotic that was not concordant with guidelines. The feedback was provided by telephone and reference to the clinical guidelines was also stated.

In total, 485 antibiotic prescriptions were analysed, 67 cases of non-adherence to the recommendations were identified, 75% (*n* = 50) corresponded to prescriptions in patients undergoing surgical procedures, 16% (*n* = 11) in the setting of skin and soft tissue infections, and the remaining 9% of inappropriate prescriptions were identified in the management of community-acquired pneumonia (*n* = 4) and intra-abdominal infections (*n* = 2). Most of the cases with inappropriate prescriptions were found in general hospitalization (82%; *n* = 55), and the antibiotics used corresponded to: cefazolin or cefepime (57%; *n* = 38), combination therapy (19%; *n* = 13), and ampicillin/sulbactam or piperacillin/tazobactam (15%; *n* = 10). Ninety-three percent of the prescribers were male (*n* = 62), with an age range of 31 to 50 years (*n* = 38), and the main reason reported for inappropriate prescribing was fear of patient deterioration or complications (67%; *n* = 45), followed by rush or lack of time (27%; *n* = 18).

These findings correlate with the results of studies conducted in other countries around the world,^5–7^ in which fear and time pressure have been identified as common barriers for appropriate antimicrobial prescribing. It is worth mentioning the limited number of studies with this approach carried out in Latin American countries, another reason to congratulate the authors of the article published in your journal,^1^ which includes data from Mexico.

However, most of the strategies for the implementation and strengthening of the AMS still fail to address these issues. Therefore, we consider it necessary to highlight to readers, who can be stakeholders in the healthcare system from different perspectives, the importance of including these aspects of beliefs and attitudes when designing educational strategies for healthcare professionals involved in the prescription of antibiotics.
